# Mediterranean Sea heatwaves jeopardize greater amberjack’s (*Seriola dumerili*) aquaculture productivity through impacts on the fish microbiota

**DOI:** 10.1038/s43705-023-00243-7

**Published:** 2023-04-24

**Authors:** Pablo Sánchez-Cueto, Orestis Stavrakidis-Zachou, Marc Clos-Garcia, Montse Bosch, Nikos Papandroulakis, Salvador Lladó

**Affiliations:** 1 LEITAT Technological Center, 08225 Terrassa, Spain; 2 Institute of Marine Biology, Biotechnology and Aquaculture, Hellenic Centre for Marine Research, 71500 Heraklion, Greece; 3 Clarivate Analytics, Barcelona, Spain; 4 Department of Genetics, Microbiology and Statistics, University of Barcelona, Av. Diagonal 643, E-08028 Barcelona, Spain

**Keywords:** Microbial ecology, Microbiome, Climate-change impacts

## Abstract

Climate change is dramatically increasing the frequency and severity of marine heatwaves (MHWs) in the Mediterranean basin, strongly affecting marine food production systems. However, how it will shape the ecology of aquaculture systems, and the cascading effects on productivity, is still a major knowledge gap. The present work aims to increase our understanding of future impacts, caused by raising water temperatures, on the interaction between water and fish microbiotas, and consequential effects upon fish growth. Thus, the bacterial communities present in the water tanks, and mucosal tissues (skin, gills and gut), of greater amberjack farmed in recirculatory aquaculture systems (RAS), at three different temperatures (24, 29 and 33 °C), were characterized in a longitudinal study. The greater amberjack (*Seriola dumerili*) is a teleost species with high potential for EU aquaculture diversification due to its fast growth, excellent flesh quality and global market. We show that higher water temperatures disrupt the greater amberjack’s microbiota. Our results demonstrate the causal mediation exerted by this bacterial community shifts on the reduction of fish growth. The abundance of members of the *Pseudoalteromonas* is positively correlated with fish performance, whereas members of the *Psychrobacter, Chryseomicrobium, Paracoccus* and *Enterovibrio* are suggested as biomarkers for dysbiosis, at higher water temperatures. Hence, opening new evidence-based avenues for the development of targeted microbiota-based biotechnological tools, designed to increase the resilience and adaptation to climate change of the Mediterranean aquaculture industry.

## Introduction

Climate change is causing an increase in the frequency and intensity of marine heatwaves (MHWs). This has been associated with mass mortality events [1]. With temperatures going up 20% faster than the global average, the Mediterranean is becoming the fastest-warming sea on our planet [2]. Improving our ability to predict the future impacts of MHWs on fish performance is of high priority, especially in aquaculture systems, one of the fastest worldwide growing food sectors, accounting for 56% of the global amount of aquatic animal food production available for human consumption [3,4].

The coastal and marine aquaculture systems are more climate-dependent than inland aquaculture, with great risk and tangible impacts caused by MHWs [5]. Increasing sea surface temperatures cause stress in farmed fish, negatively affecting key physiological aspects such as growth [6], reproductive success [7] and the immune system [8], among others. Furthermore, MHWs will impact both the water and fish microbial communities [9]. However, the knowledge on the impacts of long-lasting high-water temperatures on the water and fish microbiotas composition, and cascading effects on fish physiology and aquaculture productivity are still limited [10].

Understanding microbiota–host–environment interactions and associated ecosystem services such as food production, in the Mediterranean Sea, could contribute substantially towards achieving a more resilient aquaculture industry, by the development and uptake of novel microbiota-based biotechnological products. The gut is the most well-known mucosal area of teleosts in terms of microbial ecology, contributing to the optimization of fish digestion process [11], the production of anti-inflammatory/carcinogenic compounds and the modulation of the host immune system [12]. Those symbiotic interactions between microorganisms and farmed fishes could be disrupted due to increased temperatures, as occur in other known holobionts [13,14]. In addition, despite their closely relationship with the aquatic environment and its clear relevance on fish welfare, the skin and gills microbiota has been largely overlooked [15]. The teleost skin is composed by a complex mucus layer with immune properties [16], which depending on the health status of fishes, can encompass the microbial colonization of beneficial, commensal or pathogenic bacteria [17]. On the other hand, the fish gills are the primary site of both gas exchange and defense against pathogenic infection [18]. Several studies have been published about the influence of thermal stress in the fish gut microbiota [19–22]. However, there is a lack of holistic studies assessing the impacts of thermal stress on the fish microbiota composition and how this relates to changes in the water microbiota and fish productivity. This lack of information is especially relevant for greater amberjack (*Seriola dumerili*), a very promising fish species for aquaculture diversification in the Mediterranean [23].

The greater amberjack is a teleost from the family *Carangidae* that has been gaining relevance in the Mediterranean aquaculture due to its high market value as well as fast growth, under optimal environmental conditions [24]. Furthermore, it has a wide optimum growth temperature range (from 15 °C to 27 °C) [23]. Nevertheless, shifts in temperature, beyond its optimal growth range, are known to induce oxidative stress and metabolic changes that may be detrimental for its survival and growth, being this especially important for juveniles [6,25]. Albeit those studies are focused on greater amberjack welfare, a thorough characterization of the greater amberjack’s microbiota has never been attempted. Thus, the possible mediation of greater amberjack’s microbial community shifts caused by MHWs, on fish growth, are unknown.

To characterize and investigate the effects of MHWs on greater amberjack’s microbiota and fish physiology, we designed a MHWs simulation longitudinal study. Three temperatures were selected and implemented in a recirculating aquaculture system (RAS), where greater amberjack’s juveniles were farmed for 90 days. The control temperature (24 °C) acted as a reference point, as it corresponds to the typical summer/autumn temperatures encountered across the species farming distribution [26]. The second temperature (29 °C) was selected to provide insights on short-term climate change effects, as it represents the highest temperatures currently being recorded in the Mediterranean Sea surface, during MHWs. Finally, considering that climate models project an increase of several degrees (2–7 °C) for the Mediterranean Sea surface temperature, by the end of the century [27,28], the highest temperature (33 °C) was used to simulate future MHWs.

The present MHWs simulation study with greater amberjack’s juveniles aims to (i) characterize the microbiota composition of greater amberjack’s mucosal tissues (skin/gills/gut) at 24 °C, an optimal growth temperature; (ii) evaluate the impacts of higher, non-optimal, temperatures for greater amberjack’s farming, on the water and fish microbiota composition, in a three months MHWs simulation longitudinal study; (iii) estimate the microbial exchange between water and the fish microbiota and the effect of temperature on this exchange; (iv) assess the mediation of greater amberjack’s microbiota composition shifts, under sustained elevated water temperatures, on fish growth.

## Material and methods

### Experimental conditions

The experimental system included three identical but independent thermoregulated marine RAS, at the facilities of the certified laboratories (EL91‐BIOexp‐04) of the Institute of Marine Biology, Biotechnology, and Aquaculture (IMBBC), in Crete. Each RAS consisted of three (replicates) cylindroconical tanks (2 m^3^), connected in parallel to a water treatment system (biofilter of 2 m^3^ and a mechanical drum filter), summing up a total of nine tanks. After passing through the treatment system, water was forwarded to each tank via electric pump. From there it overflew to a common outlet which returned it to the biofilter. To provide sufficient aeration, water circulation in each tank was adjusted to 100%/h, resulting in a relative hydraulic retention time of 0.04 days. A small amount (not exceeding 15% of the total system volume per day) of saltwater from the surrounding marine area was supplied to the system. Further description of the characteristics of the tanks and the system is provided in Lika et al., 2015 [29]. Water quality parameters such as nitrogenous compounds, salinity and pH were monitored on a weekly basis, via manual measurements, and kept within safe limits for the fish (See Supplementary Table S1).

Juvenile greater amberjack (initial weight 154 ± 9 g) were obtained from the institute’s pilot scale cage farm (Souda Bay, Crete), and distributed to the nine experimental tanks (50 fish per tank). Starting from 21 °C, the real Mediterranean Sea water temperature at the beginning of the trial, the temperature at each RAS was increased by 1 °C per day until the tanks reached 24 °C (T1), 29 °C (T2), and 33 °C (T3), respectively. During the 90-days trial, the fish were fed to apparent saturation by hand, twice a day, using commercial feed (provider: IRIDA S.A.).

### Sampling procedures and DNA extraction

The MHWs simulation longitudinal study entailed three monthly (M1, M2 and M3) samplings, starting one month after the beginning of the temperature increase. At each timepoint, two fish from each tank (n = 6 for each water temperature condition) were sacrificed for microbiota sampling and weighted individually to the closest 0.1 g. For each fish, skin mucus was collected by swabbing, and subsequently the fish was dissected to obtain tissue samples from the gills and the gut. Regarding the water sampling, for each timepoint, 3 L of water was sampled from each tank (n = 3 for each water temperature condition) and filtered using nitrocellulose filters (0.2 μm pore size). All samples were immediately frozen and kept at -80^o^C. Then, total genomic DNA from the resultant 188 samples (54 skin, 54 gills, 53 gut and 27 water tank) was extracted using the ZymoBIOMICS DNA Miniprep kit (Zymo Research, Irvine, Canada). Depending on the sample type, different pre-processing methods were carried out (Supplementary Table S2), finally extracting DNA from the entire swab/filter (fish skin and water samples) or from 200 mg of the gills/gut sample.

### 16S rRNA Gene amplicon sequencing

DNA amplicon libraries were generated targeting the V3-V4 regions (341F/R805) of the 16S rRNA gene and the sequencing was performed on the Illumina MiSeq platform (PE250), following the recommendations of Illumina Inc. Sequencing was performed in Genome Québec Inc. (*Centre d’expertise et de services Génome Québec*, Montréal (Québec), Canada), as was the adapter trimming. The raw sequencing data were processed with the bioinformatic software QIIME2 version 2021.2 [30]. Briefly, pair-end reads were merged using fastq-join [31]. Chimeric sequences detection and deletion and Amplicon Sequence Variant (ASV) assignment were completed using DADA2 plugin [32]. Taxonomy was assigned at a 99% similarity level using the q2-feature-classifier plugin with the SILVA 132 database (version 2019.10.0) [33]. Then, ASVs counts table was normalized based on 16S copy numbers associated with each bacterial group [34].

### Microbial ecology and statistical analysis

The microbiota sequencing data, and its associations with the temperature and fish weight, were analyzed using the R software (Version 1.4.1717). Alpha diversity was assessed through the calculation of the Shannon index, for each type of sample, stratified by sampling month and temperature condition. For the beta-diversity, we calculated the Bray–Curtis index and also computed the corresponding centroids for the combination of temperature condition and sampling month, using the mean scores position for each type of sample group. Multivariate statistical significance analysis was carried out to evaluate the significance of the bacterial communities shifts during the longitudinal study. One-way PERMANOVA was performed with the *Adonis* R package (999 permutations, *p* < 0.05) to assess the effect of time on beta diversity, for the control water temperature. Two/three-way PERMANOVA was performed with the *pairwise Adonis* R package (999 permutations, *p* < 0.05) to compare the effects of temperature and/or time for the different types of sample (water, fish skin, gills and gut). Taxonomical differences were computed both at phylum and genus levels. To do so, we transformed the ASVs counts with the centered log-ratio (CLR) approach, to assess the compositionality of the data [35]. Thus, differential abundance analyses for bacterial genera were performed by ANOVA with Tukey HSD post-hoc test, using the CRL data. The software *Fast expectation maximization for microbial source tracking* (FEAST) [36] was used to estimate the microbiota exchange between the different types of sample (water, fish skin, gills and gut), for the three temperatures studied. For this estimation, the ASVs counts per sample, previously inferred by the DADA2 plugin, were used as input. The samples were organized to assess the contribution to the mucosal microbiotas’ composition (gut, gills or skin) by the different microbial sources (gut, gills, skin, water tank and the same type of sample from the previous month). The contribution of the microbial sources into the sinks was calculated by an expectation-maximization algorithm implemented in the FEAST software [37]. Shared ASVs between the different types of samples were identified through the UpSet R package.

Finally, a causal mediation approach [38] was used to unravel the associations between the water temperature, the gut microbiota composition, and the fish growth, considering the temperature as the exposure variable, the final fish weight as the response, and the gut microbiota as the mediator. For the gut microbiota composition (mediator), the score values of the two first principal coordinates derived from beta diversity PCoA were used. Spearman correlations between individual ASVs abundance, at M3, and fish growth (weight increase) were carried out using the *corr* R package.

## Results

### Greater amberjack´s microbiota composition at water control temperature

**Fig. 1:**
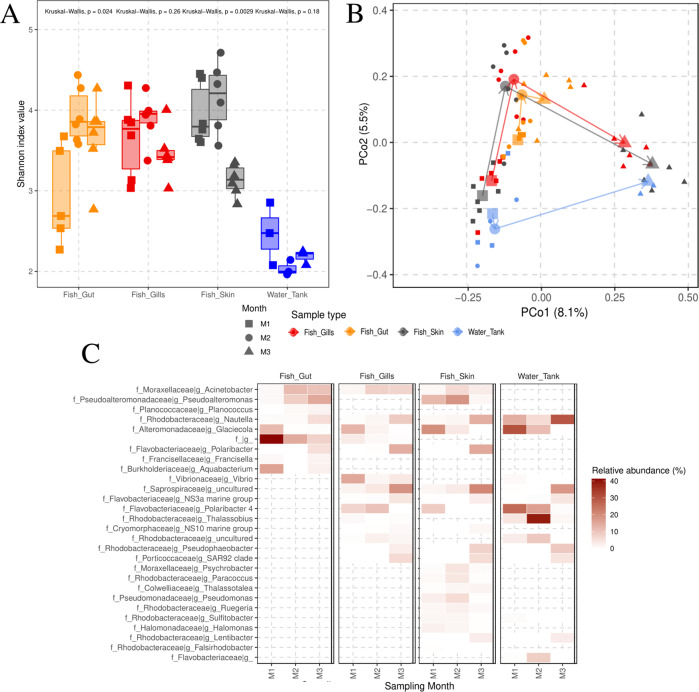
General characterization of greater amberjack’s microbiota. **A** Alfa-diversity distribution analysis for the different types of samples assessed: skin, gills, gut and water. For each type of sample, at each of the selected timepoints, the individual Shannon value is represented by points, and the niche’s richness distribution, as a boxplot, with the median of the distribution indicated as a horizontal bar. Kruskal–Wallis test is presented for each sample type, indicating a significative change over time (*p* < 0.05). **B** Beta diversity PCoA, computed upon the Bray–Curtis distance matrix, displaying fish and water samples from the control temperature tanks (24 °C). Samples from different timepoints are shown in different shapes: squares for Month 1 (M1), circles for Month 2 (M2) and triangles for Month 3 (M3). Each sample type (fish skin, gills, gut and water) has been colored differently. For each combination of samples belonging to the same type and month, we computed and plotted the centroid of the corresponding cluster. Afterwards, centroids belonging to the same sample type have been joined sequentially depending on the sampled month (M1 - >M2 - >M3), to show the trajectory of the bacterial community over time. Explained variance for each of the coordinates is indicated between parentheses next to the corresponding axis. **C** Heatmap displaying the relative abundance (%) of those bacterial genera found to be differentially abundant, in greater amberjack and water bacterial communities, in relation to the sampling timepoint. Significance was assessed with an ANOVA test upon the CLR-transformed genera counts, comparing between the three months treatment.

The bacterial communities of the greater amberjack mucosal tissues were significantly more diverse than those of the surrounding water (Fig. 1A, Supplementary Table S3) throughout the study, at T1. However, the Shannon index showed different trends depending on the type of tissue. While the diversity of bacterial communities from gut samples significantly increased over time, the opposite trend was observed for the skin and gills microbiota. However, the decrease in bacterial diversity was only statistically significant for the skin samples.

In terms of beta diversity (Fig. 1B), the bacterial communities in all three different mucosal tissues, and the water tank, at T1, significantly changed over time (PERMANOVA, *p*-value: skin = 0.001, gills = 0.001, gut = 0.001, water = 0.005). At the beginning and at the end of the study, the beta diversity of the outermost mucosal tissues and the surrounding water was statistically non different, and, at the same time, significantly different from the gut microbiota (Supplementary Table S4).

The greater amberjack microbiota was composed at M1 by 23 bacterial phyla and 348 genera. Overall, the bacterial communities from greater amberjack and the water tank, at M1, were dominated by the phylum Proteobacteria (Gut = 45.4% (+/−17.5), Gills = 73.2% (+/−11.7), Skin = 77.1 (+/−2.1), Water = 70.5% (+/−2.3)) and Bacteroidetes (Gut = 7.4% (+/−9.3), Gills = 15% (+/−7.1), Skin = 16.1% (+/−2.5), Water = 29% (+/−2.1)). At the end of the study, a significant increase of Bacteroidetes in skin, gills, and water tank was detected, (*p* < 0.05) mainly driven by the genera *NS3a marine group*, *Polaribacter* and an unidentified genus from the family *Saprospiraceae* (Fig. 1C). However, this significant increase in the relative abundance of genera belonging to the phylum Bacteroidetes was not detected in the greater amberjack’s gut. In the gut, the genera that gained more importance in terms of relative abundance during the longitudinal study, generally corresponded to the phylum Proteobacteria, such as *Acinetobacter*, *Pseudoalteromonas* and *Nautella*. The genus *Glaciecola* decreased its relative abundance in all the types of samples studied.

### Greater amberjack and water microbiota dynamics under two MHW simulated conditions

Shannon index values corresponding to the water tanks’ bacterial communities in T2 (29 °C) and T3 (33 °C) showed the same dynamics than for T1 (24 °C), with significantly lower alfa diversity than greater amberjack’s mucosal tissues along the whole longitudinal study (Supplementary Fig. 2, Supplementary Table S3). At the end of the study (M3), when comparing the Shannon index at different temperatures (T1-T2-T3), we found no significant differences between alfa diversity from the bacterial communities present in the different types of samples, except for the gut microbiota, where alfa diversity is significantly lower at T3 in relation to T1.

**Fig. 2:**
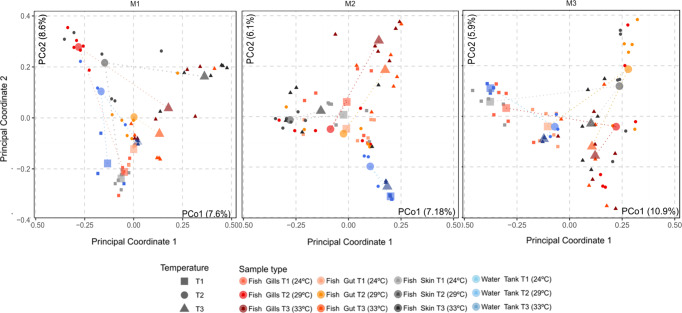
Beta diversity progression of greater amberjack and water microbiotas throughout marine heatwaves simulation in RAS systems. Beta diversity PCoA, computed upon the Bray–Curtis distance matrix, divided by each sampling timepoint (M1, M2, M3, from left to right). Fish and water samples, from the three water temperatures assessed in the study, are shown in different shapes: squares for 24 °C (T1), circles for 29 °C (T2) and triangles for 33 (T3). Each sample type has been colored differently. For each combination of samples belonging to the same type and temperature, we computed and plotted the centroid of the corresponding cluster. Afterwards, centroids belonging to the same sample type have been joined sequentially depending on the sampled month (T1–T2–T3).

In terms of community structure, the beta diversity of the water microbiota was not statistically affected by temperature. On the other hand, greater amberjack’s microbiota suffered different statistically significant shifts in beta diversity, depending on the mucosal tissue and timepoint (Fig. 2, Supplementary Table S4). At T3, bacterial community composition from all greater amberjack’s tissues (skin, gills and gut) were statistically different compared to T1, throughout all time points. The same happened for T2 at M1 and M3, but at M2 significant differences were just observed in skin and gills. When comparing beta diversity between T2 and T3, the gut bacterial communities were significantly different at the three timepoints. On the contrary, at M3, for T2 and T3, respectively, bacterial community structure from skin, gills and gut was not significantly different.

**Fig. 3:**
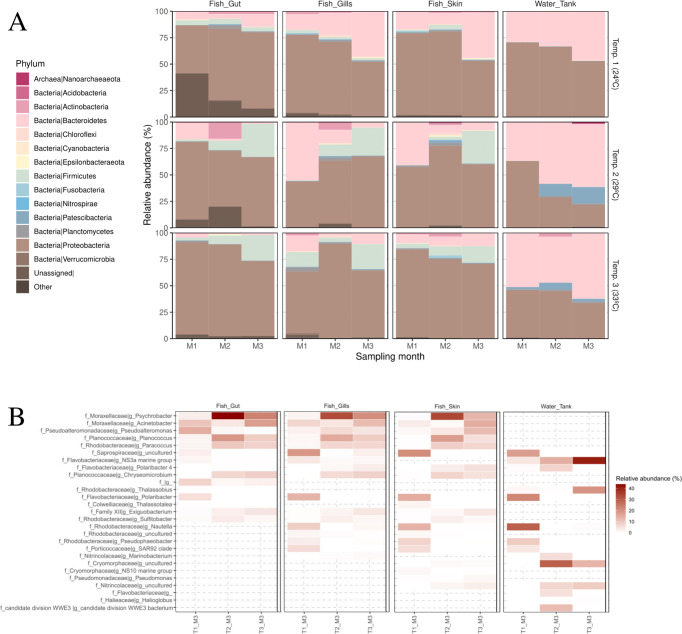
Taxonomical shifts in greater amberjack and water microbiotas throughout marine heatwaves simulation in RAS systems. **A** Bar graph showing the taxonomical composition of the bacterial communities from the different fish mucosal tissues and the water tanks (M1-M3 and T1–T3), at the phylum level (mean relative abundance). Within each bar, different colors were assigned to the 15 most abundant phyla, grouping the remaining ones in the category “Other”. For the complete composition profile, see Supplementary Table S5. **B** Heatmap displaying the mean relative abundance (%) of those genera that were found to be differentially abundant in relation to each of the three temperature categories (T1–T2 –T3). Significance was assessed with an ANOVA test upon the CLR-transformed genera counts.

At phylum level, the greater amberjack’s bacterial composition differences observed between samples at T2 and T3, compared to T1, were generally mediated by the increase of Proteobacteria and Firmicutes phyla in all three tissues (skin, gills and gut), especially at end of the longitudinal study (Fig. 3A, Supplementary Table S5). Particularly in the gut, at M3, the differential abundance analysis, at the genus level, showed the increase in relative abundance (T2 and T3 compared to T1) of the genera *Psychrobacter*, *Chryseomicrobium, Planococcus, Planomicrobium*, *Paracoccus* and *Polaribacter 4*. This contrasted with the significant decrease of ASVs corresponding to *Pseudoalteromonas, Nautella, NS3a* marine clade, *Polaribacter* and an uncultured genus from the family *Saprospiraceae* (Fig. 3B, Supplementary Tables S6 and S7). At T3, it is remarkable the high relative abundance of *Enterovibrio* in the greater amberjack’s gut samples from 4 out of 18 individuals analyzed, at T3, (82–97% of relative abundance).

In relation to the water tanks, their microbiota at T2 and T3, compared to T1, showed an increase in the dominance of Bacteroidetes and a significant appearance of ASVs belonging to the phylum Patescibacteria, while Proteobacteria decreased its abundance (Fig. 3A). Thus, higher temperatures significantly promoted the abundance of *NS3a marine group* and the *Cryomorphaceae* family. Whereas, in both T2 and T3, significantly decreased the abundance of *Nautella*, *Polaribacter* and *Saprospiraceae* family, compared to T1 (Fig. 3B).

We further investigated the potential transference of bacterial ASVs between the water and the greater amberjack’s microbiota (Supplementary Fig. S3, Supplementary Table S8). In general, the contribution of the water microbiota in the fish microbiota was limited across the longitudinal study. At the end of the study (M3) (Supplementary Fig. S3C), we only observed a moderate contribution of the water tank microbiota into the skin microbiota (FEAST: 35%), whereas a very limited contribution occurred at T2 and T3 (FEAST: 4% for both temperatures). In terms of shared ASVs, at M3 for T1, the skin microbiota shared 35 ASVs with the water microbiota. This 35 ASVs summed up a 55% of the total relative abundance of bacteria present in the fish skin. On the contrary, at M3, for T2 and T3, the skin microbiota shared with the water tanks 22 and 8 ASVs, respectively, accounting for an 8% and 4% of the total relative abundance (Supplementary Table S9a).

### Impacts of water temperature on fish microbiota composition and growth

**Fig. 4:**
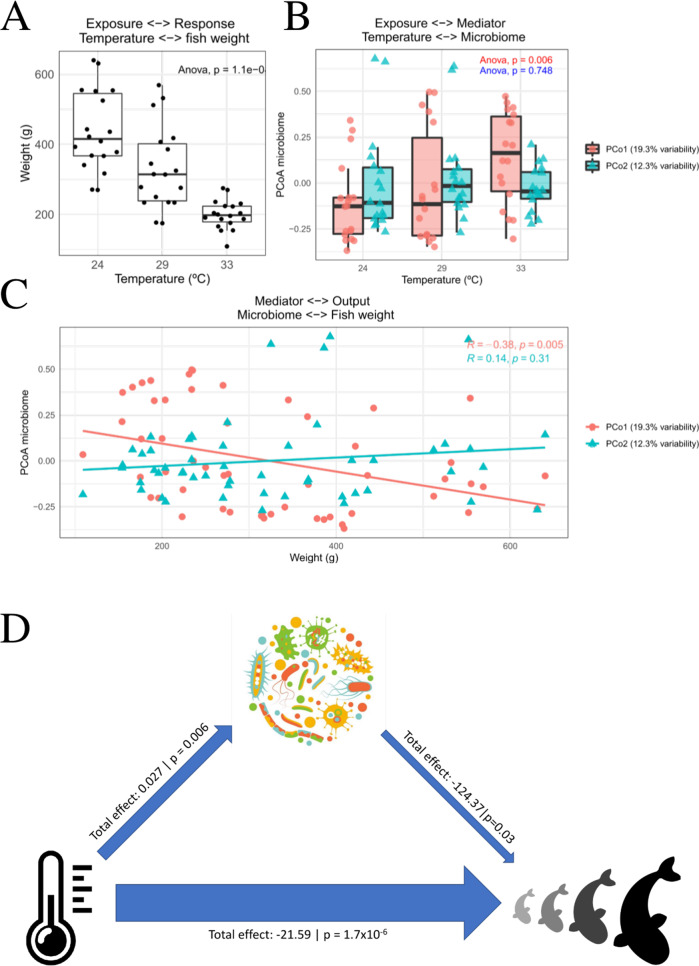
Causal mediation analysis (CMA) dissecting the total direct effect of temperature on greater amberjack’s growth, and the indirect effect mediated through the greater amberjack’s gut microbiota composition. **A** Association between the water temperature and the fish weight. **B** Associations between the scores values of the two first coordinates derived from the PCoA transformation of the Bray–Curtis distance matrix and the water temperature. **C** Correlations between the microbiota, summarized as the scores values of the first two beta diversity coordinates, and the fish weight. **D** Summary of the mediation analysis. The design of the analysis was to consider temperature as the exposure, fish weight as the response and the gut microbiota, summarized as the first beta diversity coordinate, as the mediator. Each arrow indicates the total effect between each pair of variables, including its *p*-value. Arrows width indicates the effect size of each interaction.

Along the longitudinal study, greater amberjack’s weight increased at T1 (+346 g; mean value) and T2 (+320 g; mean value), while at T3 growth was significantly lower (+46 g; mean value) (Supplementary Table S10) and significantly compromised by temperature (*p* = 9.1e−07) (Fig. 4A).

The same mediation analysis showed that the association between the temperature and greater amberjack’s gut microbiota composition (beta diversity) is captured in the first coordinate (PCoA1 *p* = 0.006), while discarded in the second (PCoA2 *p* = 0.748) (Fig. 4B). Regarding greater amberjack’s gut microbiota beta-diversity and weight, a significant negative association between the first coordinate and the fish weight was observed, while no association was found for the second coordinate (Fig. 4C).

The mediation analysis revealed that the total effect of temperature on the fish growth was of −21.59 g/°C (Fig. 4D). Our analysis showed that the gut microbiota composition explained around 16% of this temperature effect upon the fish weight, being its contribution statistically significant (*p*-value 0.026). More in-depth, correlation analysis showed that ASVs, found in the fish gut, and belonging to the genera *Pseudoalteromonas* (*R* = 0.5; *p* = 0.036/*R* = 0.47; *p* = 0.05), and an uncultured one from the Gammaproteobacteria class (*R* = 0.55; *p* = 0.019), are positively correlated with fish performance, whereas ASVs belonging to the genera *Paracoccus* (*R* = −0.52; *p* = 0.028) and *Chryseobacterium* (*R* = −0.48; *p* = 0.045) present a negative correlation between their relative abundance and fish growth. Although two other ASVs belonging to the *Psychrobacter* genus were significantly correlated with fish growth, one correlated positively and the other negatively (Supplementary Table S9b).

## Discussion

To the best of our knowledge, we provided the first description of the bacterial communities present in the skin, gills and gut from juvenile greater amberjacks, in a longitudinal study under optimal growth temperature (24 °C). Those communities had higher alpha diversity than the surrounding water, which showed low alpha diversity values at all timepoints. Lower diversity values in the water tanks can be caused by working in closed systems. RAS systems have been described as low bacterial diversity environments, with higher microbial carrying capacity than the intake water, due to an increased organic load from fish feed and faeces and a high hydraulic retention time [39]. This was also true for our RAS system, where the bacterial diversity found in the tanks for the three timepoints and temperatures was significantly lower than the water inlet (Supplementary Table S11). However, closed systems are the only feasible option to accurately control and manipulate environmental parameters such as temperature, when facing the challenge to simulate MHWs [40,41]. Thus, being key for researchers in the field of microbial ecology to take into consideration this limitation when extracting conclusions from the results.

On the other hand, in terms of beta diversity, the greater amberjack’s skin and gills presented more similar bacterial community structure with the surrounding water than with their own gut samples, at the different selected timepoints. This niche-specific development is coincident with other teleost studies, where the microbiotas in outermost mucosal surfaces were more similar to the water column than the innermost parts, such as the gut, due to its continuous state of interaction [42,43]. In addition, the shifts in microbial composition in mucosal tissues over our three-month study period, at the control temperature, can be attributed to the host selection pressure that has been observed in other species [44–46]. Yet, other studies have shown that only the most abundant phylum (Proteobacteria) was shared between fish skin and gills and the surrounding water [47]. These controversies reinforce the need for further studies that consider the relationships between environmental (water) and fish skin and gills microbiota in aquaculture systems.

In this direction, the FEAST analysis indicated a lower contribution of the water microbiota on the fish and gills bacterial diversity than expected, when observing the relative abundance of genera present in the water and fish microbiota, during the longitudinal study at T1. To double-check and go beyond the FEAST results, we assessed the number of ASVs shared between the water and the skin microbiota, and their relative abundance. Our results show that, at the end of the experiment, for the control temperature tanks, the relative abundance of the shared ASVs between the water tank and the fish skin, mostly belonging to the genera *Polaribacter* and *Nautella* or the family *Saprospiraceae*, accounted for more than the 50% of the total relative abundance of bacteria present in the skin, a higher percentage than the FEAST output. Thus, suggesting the importance to combine both methodologies to get a better understanding of the importance of the environmental microbiota as a source of microbial biodiversity in animal tissues.

In the case of the greater amberjack’s gut microbiota, we could see a differentiation of the community in respect to the outer fish tissues developing with time. These differences were driven by an increased abundance of genera such as *Polaribacter*, *NS3a marine group* and unidentified *Saprospiraceae*, in the greater amberjack’s skin and gills. This observation may be related to their capacity of biofilm development in fish mucosal tissues, previously reported for different genera in the Bacteroidetes phylum in similar environments [48] and consistent with observations reported for other fish species [49,50]. Besides Bacteroidetes, we observed that members of the genus *Pseudoalteromonas* (Proteobacteria) increased their abundance in the greater amberjack’s gut microbiota across time, while decreasing their abundance in the rest of the mucosal tissues and the water tank.

In addition to the longitudinal study at 24 °C, in the present work we also show that increasing water temperatures, simulating marine heatwaves at 29 °C and 33 °C for three months, highly influenced the fish microbiota’s composition. The microbial diversity found in greater amberjack’s gut samples at T3 was significantly lower than those at T1, from M2 and onwards. This result is consistent with rainbow trout studies where after the exposure to high water temperatures, the gut microbiota alpha diversity decreased [20,22,51]. Although there is no clear consensus between the effects of temperature on fish gut alpha diversity, reduction of microbial richness could increase the risk of microbiota imbalance (dysbiosis), leading to changes in bacterial community functionality and negative interactions with the host immune system [52,53].

Shifts observed in the greater amberjack’s skin, gills, and gut beta diversity, in the two RAS systems simulating increasingly severe MHWs, were significantly different to the bacterial dynamics found in the surrounding water, and also the bacterial community shifts observed at T1. Thus, confirming the relationship between water temperature and microbiota composition. Microbiota community shifts were more evident for the skin and gills, which may be attributed to the chemical and physical alteration of the mucus layer caused by thermal stress, considering that the skin and gills of teleost fish are a dynamic interface in constant contact with the environment [16,54], and the fish tissues most affected at physiological level by thermal variability [55]. In this sense, both methodologies, FEAST and the quantification of the relative abundance of the shared ASVs between the water and the fish skin microbiota, suggest that MHWs have the potential to highly disturb the ecological equilibrium between the water and fish bacterial communities, leaving room for opportunistic bacteria to colonize the outer fish tissues.

When we focused on the bacterial diversity dynamics between the three different fish tissues, at high water temperatures, we observed an increase in abundance of Firmicutes in T2 and T3 in the skin, gills and gut microbiota, producing and homogenization of bacterial community structures between inner and outer mucosal tissues, not observed at T1. These shifts were significantly different from the water microbiota dynamics, where Bacteroidetes increased its dominance in relation to Proteobacteria.

The observed increase of the Firmicutes phylum in the greater amberjack’s microbiota was caused by the increase in abundance of the ASVs corresponding to the genus *Planococcus*, previously reported in fish microbiotas [56]. Other genera that increased their abundance with temperature after three months in all greater amberjack’s mucosal tissues were *Psychrobacter*, and at less extent, *Chryseomicrobium*, *Paracoccus* and *Polaribacter 4*. The genus *Psychrobacter* is described as a highly stress and temperature tolerant bacterium with capacity to growth at temperatures between −10 °C and 42 °C [57]. Albeit some members of the *Psychrobacter* have been reported as opportunistic pathogen species in fish and humans [58,59], others were also described by its antagonistic activity against fish pathogens [60], and were even used as probiotics for fish [61,62]. This controversial literature in relation to the possible detrimental or beneficial effects of isolated members of particular bacterial genera, reinforce the opinion that for each studied ecosystem, to better understand its microbial ecology and the metabolic potential of the inhabitant microbes, tailor-made isolation studies would be of high value.

In this sense, the relationship between water temperature, fish microbial diversity and fish growth is one of the main topics of study in aquaculture [63,64]. Previous studies claim that out of the optimal fish temperature, growth and survival rate may be compromised [65]. Our results are consistent with these previous works. However, the novelty of our work derives from the assessment of the role of the microbiota as a mediator between the exposure of the fish to high temperatures and the impact it may have on its growth. Other studies have shown the effect of rising water temperature on the fish microbiota composition [66,67] or growth [25,68], but there are no studies correlating impacts of water temperature on the microbiota of a major aquaculture specie and its implications for growth and, thus, for aquaculture productivity.

Recent microbial ecology research has produced high evidence that, in most of fish species, gut microbiota has a host-specific composition and plays a critical role in nutrient uptake and health maintenance [69]. Recently, several studies in animal species, suggested that changes in gut microbiota composition due to thermal stress may have physiological consequences for the host [70]. In our study we contributed to this research by demonstrating the impact of the exposure to simulated MHWs on the greater amberjack’s gut microbiota composition, and the mediation it exerts on the negative cascading effects for fish growth, as has been demonstrated by the results of the mediation analysis. Hence, concluding that the gut microbiota composition was modified by the temperature and that both the temperature increase, and the specific microbiota composition resulting of this increase, negatively affected the fish weight and, therefore, the aquaculture system productivity. This is in line with the proposed adaptation of the Koch’s postulates by Byrd and Segre [71]. In their case, they highlighted the importance of microbial communities in modifying disease outcome, providing a nuanced view of strict causation. In the present work, we want to highlight the importance of microbial communities to modulate the impacts of environmental (and/or biotic) stressors on macroorganisms. This is crucial in the case of climate change and how will impact marine food production systems. MHWs simulations resulted in greater amberjack’s microbiota alterations and compromised growth.

Beyond the structure of whole bacterial communities, our results from the differential abundance analysis at the genus level, and the correlation analysis between ASVs and fish growth at different temperatures, suggest members of the genera *Psychrobacter, Chryseomicrobium, Paracoccus* and *Enterovibrio* as potential dysbiosis biomarkers in aquaculture, in relation to MHWs [72]. ASVs belonging to the *Enterovibrio* genus were only found at high abundance in the gut of the greater amberjack’s juveniles at T3. This genus includes well-known opportunistic pathogen species presents in fish mucosal areas [17,73], and is correlated with high water temperatures in other fish species [19]. The description of bacterial ASVs enriched under increased water temperatures, and negatively correlated with fish performance, makes them good target candidates to open new avenues for phage therapy, a field of research still in its infancy in relation to aquaculture.

Our results also support the genus *Pseudoalteromonas* as a potential biomarker for healthy and resilient aquaculture systems. *Pseudoalteromonas* has been positively correlated with good fish feed efficiency [74] and some strains are used as probiotic in fish farming to immobilize pathogenic *Vibrio* strains [75]. Thus, highlighting the potential of *Pseudoaltermonas* strains not only as probiotics for present day aquaculture [76], but also as new tailor-made biotechnological products for increasing the resilience of the aquaculture industry to future climate change impacts.

However, it is important to underline that our results were obtained in a controlled environment. Further research attempts will require an improvement of microbial diversity monitoring in open sea aquaculture facilities. Hence, collecting water and greater amberjack’s samples during real MHWs in the Mediterranean. This would be key to confirm our results in a real environment, and also to test and prototype novel probiotics, symbiotics or phage therapy bioproducts for a resilient aquaculture to climate change.

## Supplementary information

Supplementary Table S1

Supplementary Table S2

Supplementary Table S3

Supplementary Table S4

Supplementary Table S5

Supplementary Table S6

Supplementary Table S7

Supplementary Table S8

Supplementary Table S9

Supplementary Table S10

Supplementary Table S11

Supplementary figures

## Data Availability

The datasets generated and/or analyzed during the current study are available in the European Nucleotide Archive (ENA) at EMBL-EBI with accession number PRJEB56519. Supplementary information: The trial was performed according to the legal regulations (EU Directive 2010/63) and upon its approval by the regional veterinary authorities and the Ethics Committee of the IMBBC (Ref number 255,344). Juvenile greater amberjack for the trial were obtained from the institute’s pilot scale cage farm (Souda Bay, Crete).
