# Supplementary figures and images for: Mediterranean Sea heatwaves jeopardize greater amberjack’s (Seriola dumerili) aquaculture productivity through impacts on the fish microbiota
Source: ISME Commun. 2023 Apr 24;3:36. doi: 10.1038/s43705-023-00243-7 (PMC10125963; doi:10.1038/s43705-023-00243-7)

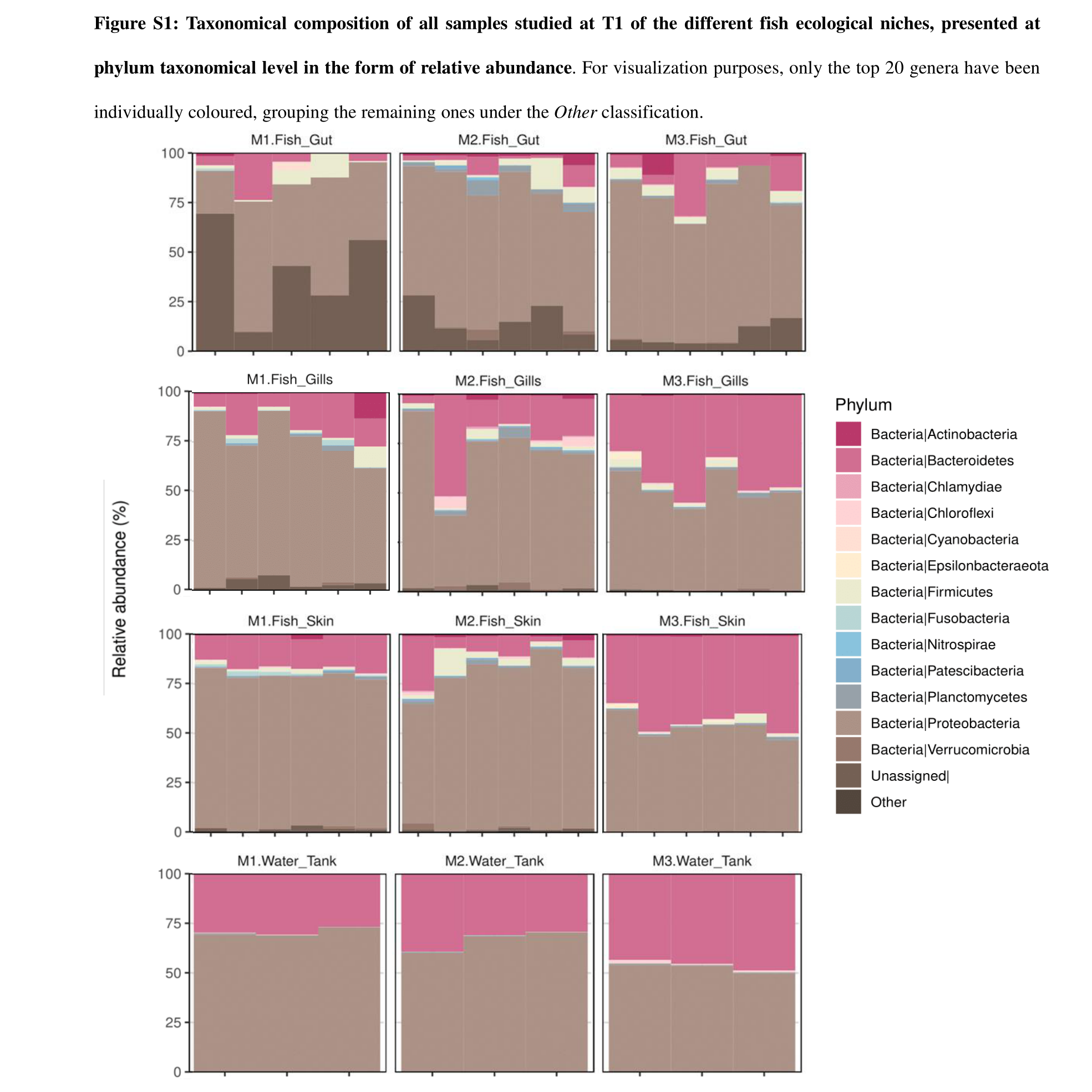

Supplement: Supplementary file 12 — Supplementary figures [file 43705_2023_243_MOESM12_ESM.tif]
